# Bioactivation Routes of Gelatin-Based Scaffolds to Enhance at Nanoscale Level Bone Tissue Regeneration

**DOI:** 10.3389/fbioe.2019.00027

**Published:** 2019-02-15

**Authors:** Maria Grazia Raucci, Ugo D'Amora, Alfredo Ronca, Christian Demitri, Luigi Ambrosio

**Affiliations:** ^1^Institute of Polymers, Composites and Biomaterials, National Research Council, Naples, Italy; ^2^Department of Engineering for Innovation, University of Salento, Lecce, Italy

**Keywords:** scaffold, natural polymer, bone tissue engineering and regeneration, BMP-2 peptide, hydroxyapatite

## Abstract

The present work is focused on the development of gelatin-based scaffolds crosslinked through carbodiimide reaction and their bioactivation by two different methods: (i) surface modification by inorganic signals represented by hydroxyapatite nanoparticles precipitated on scaffold through biomimetic treatment; (ii) analog of BMP-2 peptide decoration. The results showed the effects of polymer concentration and crosslinking time on the physico-chemical, morphological, and mechanical properties of scaffolds. Furthermore, a comparative study of biological response for both bioactivated structures allowed to evaluate the influence of inorganic and organic cues on cellular behavior in terms of adhesion, proliferation and early osteogenic marker expression. The bioactivation by inorganic cues induced positive cellular response compared to neat scaffolds in terms of increased cell proliferation and early osteogenic differentiation of human mesenchymal stem cell (hMSC), as evidenced by the Alkaline phosphatase (ALP) expression. Similarly BMP-2 peptide decorated scaffolds showed higher values of ALP than biomineralized ones at longer time. The overall results demonstrated that the presence of bioactive signals (either inorganic or organic) at nanoscale level allowed an osteoinductive effect on hMSC in a basal medium, making the modified gelatin scaffolds a promising candidate for bone tissue regeneration.

## Introduction

In bone tissue engineering, highly porous three-dimensional (3D) scaffolds play a critical role in new tissue formation for their similar structure to natural bone tissue. Indeed, the function of scaffold should be to provide a 3D spatial and temporal structure to guide cell infiltration and proliferation, leading to a new tissue (Kim et al., 1991). This purpose may be achieved with an open porosity that allows cell migration, growth and nutrient transport, providing in the same time a good mechanical support for new tissue (Weineir and Wagner, 1998; Kelly and Prendergast, 2005).

Some natural polymers as polysaccharides (Zerwekh et al., 1992; Demitri et al., 2016; D'Amora et al., 2018b) (i.e., alginate, chitosan, cellulose, hyaluronic acid) and proteins (i.e., gelatin and collagen) have been widely used for scaffold preparation (Timkovich, 1977), due to their biocompatible and biodegradable properties. In this study, a biodegradable and biocompatible protein such as gelatin was chosen for scaffold development, for its not antigenicity in physiological environment if compared to collagen (Liang et al., 2004). Furthermore, its physico-chemical performances can be appropriately modulated.

The main limitation of gelatin polymer is its high biodegradation rate due to enzymatic digestion and high solubility in physiological environment, determining a poor mechanical stability that leads to a mismatch between the new bone formation and the scaffold degradation rates. However, chemical treatments may be performed to improve the degradation time due to the presence of active chemical groups (NH_2_ and COOH). In this context, some chemical crosslinkers as genipin, glutaraldehyde (GTA) and diisocyanates, as well as carbodiimides and polyepoxy compounds can be used (Kang et al., 1999).

Among all, carbodiimide is a linker agent used between carboxylic acid and amino groups to form amide-type bonds (LeGeros, 1981). The crosslinking of gelatin hydrogel with carbodiimide involves the activation of its carboxylic acid groups of glutamic or aspartic acid residues to give *O*-acylisourea groups, which form crosslinks after a reaction with its free amino groups of lysine or hydroxyline residues (Jarcho, 1981; LeGeros, 1981). The crosslinked structure results in a lower degradation rate, increasing the longevity of materials in the living environment. Furthermore, the presence of chemical groups on the polymer chain allows the bioactivation of scaffolds by specific signals able to trigger the cellular behavior in terms of proliferation and osteogenic differentiation of human cells. To this purpose, biomimetic functional scaffolds can be developed by modifying them with bioactive signals including inorganic nanoparticles (i.e., hydroxyapatite) and organic compounds such as osteogenic growth factors/peptides (Neuman and Neuman, 1958; Kim et al., 2004; Dessì et al., 2012). These types of scaffold modifications provide biochemical cues for promoting stem cell osteogenic commitment. In the present paper, two different bioactivation routes of gelatin-based scaffolds were pursued through an easier approach which concerns the functionalization with organic and inorganic signals, to enhance at nanoscale level bone tissue regeneration. First, a bioactivation by inorganic nanoparticles was performed through biomimetic approach which allows the deposition of crystalline hydroxyapatite nanoparticles on scaffold surface. In this way, the surface changes in terms of roughness allow an improvement of cell attachment and the chemical composition acts as inductive factor for osteogenic differentiation (Demitri et al., 2016). The biomimetic approach is based on the deposition of hydroxyapatite nanoparticles through two steps of treatment in a simulated body fluid solution (SBF) at different incubation time. The apatite formed in SBF is considered to exhibit higher bioactivity and biocompatibility (Cortesi et al., 1998; Broderick et al., 2005; Catauro et al., 2007) than sintered hydroxyapatite.

Moreover, gelatin scaffold was functionalized also by an organic signal as peptide mimicry growth factors which was covalently immobilized to the scaffold through carbodiimide reaction. Here a peptide, characterized by 20 amino acids corresponding to a 68–87 sequence in knuckle epitope of BMP-2, was modified by adding an amide group to the end of sequence and successively it was covalently linked to carboxyl group of gelatin scaffold. It is well-known that BMP-2 peptide shows *in vivo* and *in vitro* osteogenic activity when immobilized on polymeric scaffolds as alginate (Saito et al., 2004) and chitosan (Soriente et al., 2018). In this study, the effect of crosslinking time on gelatin scaffold performances, in terms of swelling and degradation, was evaluated. Furthermore, the effect of inorganic functionalization by biomimetic approach on mechanical properties and on *in vitro* biological behavior was evaluated through proliferation and early osteogenic differentiation studies by using human mesenchymal stem cells (hMSC). The gelatin scaffold at specific composition and crosslinking time, with improved performances, was chosen for the organic functionalization by using amide-modified BMP-2 peptide. In this way, the effect of inorganic and organic cues on cellular commitment was evaluated.

## Materials and Methods

### Materials

Gelatin type B (bovine skin, 225 Bloom), 1-ethyl-(3-3-dimethylaminopropyl carbodiimide hydrochloride) (EDC) and all reagents used to prepare SBF solution: Calcium chloride (CaCl_2_), Magnesium chloride hexahydrate (MgCl_2_·6H_2_O), Sodium bicarbonate (NaHCO_3_), Potassium hydrogen-phosphate trihydrate (K_2_HPO_4_·3H_2_O), Sodium sulfate anhydrous (Na_2_SO_4_), Potassium Chloride (KCl), Sodium chloride (NaCl) were purchased from Sigma-Aldrich (Milano, Italy).

### Preparation of Crosslinked Gelatin Scaffolds

Type B Gelatin was dissolved in deionized water (dH_2_O) (5–10 wt/v%, named B5 and B10, respectively) at 40°C, rpm 100. After 30 min of stirring, the solutions were sonicated to remove air bubbles and then poured into a Teflon mold to be processed for 48 h by freeze-drying. The crosslinking of Gelatin was performed by soaking porous lyophilized scaffolds, at different time points (1, 3, and 6 h) at room temperature, in acetone–water solution (4:1 v/v) containing a water-soluble EDC, followed by incubation at 4°C for 24 h. The scaffold/solvent volume ratio was kept at 1 wt/v%, considering our previous study (Raucci et al., 2018), in order to maintain a porous structure. Meanwhile, the amount of crosslinking agent was 0.7 wt/v% respect to volume solution of acetone-water (Raucci et al., 2018). The crosslinked scaffolds were washed several times in dH_2_O to remove some EDC residues and then dehydrated in ethanol solutions.

### Scaffold Bioactivation Procedure

#### 3D Scaffolds Coding System

For presentation clarity, a coding system to discriminate between all the fabricated scaffolds will be used throughout the text.

In the case of untreated scaffolds, the following coding system will be employed: “BX_Yh,” where “X” is the gelatin concentration (5 or 10 wt/v%) while “Y” is the crosslinking time (1, 3, or 6 h).

In the case of functionalized scaffolds, the adopted nomenclature will be: BX_Yh/A, with X and Y defined as before, while A is: (i) “bio” (biomimetic treatment) and (ii) “BMP” (organic functionalization).

#### Biomimetic Surface Treatment

To obtain biomineralized scaffolds with bioactive solid signals on the gelatin scaffold surfaces, a simple biomimetic treatment (Abe et al., 1990; Tanahashi et al., 1994; Kim et al., 2005) was performed by using simulated body fluid solutions (5 × SBFs). The treatment is based on two steps with different SBF solutions (5 × SBF_1_ and 5 × SBF_2_) at different pH values (pH = 6.5 and 6.0), to stimulate the hydroxyapatite nuclei formation (3 days) and crystallization (4 days), respectively, as reported in our previous work (Soriente et al., 2018). The biomimetic scaffolds were gently rinsed in dH_2_O to remove excess ions and then dried overnight under laminar hood.

#### BMP-2 Peptide Covalent Immobilization

**Figure 1:**
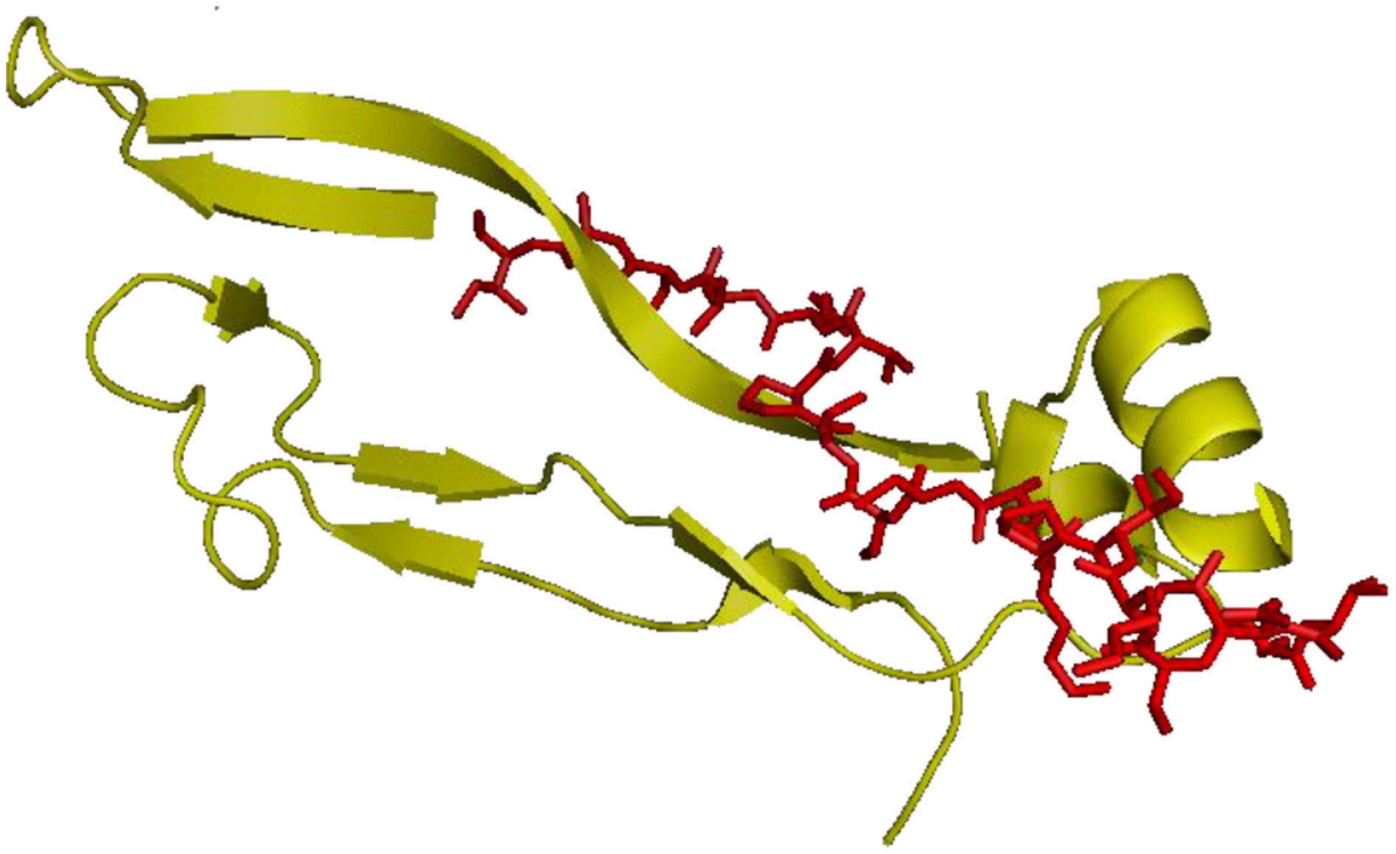
hBMP-2, Knuckle Epitope, residue 68–87. In red the modified BMP-2 peptide sequence (H-NSVNSKIPKASSVPTELSAI-amide).

The organic functionalization of gelatin–based scaffolds was performed by covalent immobilization of BMP-2 like-peptide. The amide-modified peptide sequence (H-NSVNSKIPKASSVPTELSAI-amide) is reported in Figure 1. The synthesis was performed by microwave-assisted Fmoc solid phase peptide technology as showed in a previous study (Soriente et al., 2018).

The peptide was characterized by analytical High Performance Liquid Chromatography (HPLC, Agilent) and mass spectrometry (micro-TOF; Bruker), before and after purification by preparative HPLC. Furthermore, the covalent immobilization by EDC:NHS (molar ratio 1:0.2) took place between amide group of peptide and gelatin COOH group (COOH: EDC: NHS: BMP-2 = 1: 0.5: 0.1: 0.0006). The amount of BMP-2 peptide used for bioactivation was 1.8 μg/1.0 mg of scaffold.

## Characterization

### Swelling and Degradation Investigations of Neat Gelatin Scaffolds

The swelling and degradation measurements were carried out on dried crosslinked scaffolds (height = 9 mm, diameter = 6 mm) immersed into 4 mL of dH_2_O at 37°C. At defined time points, swollen hydrogels were weighed after removal of water in excess with a filter paper. Meanwhile for degradation study, scaffolds were frozen (−86°C) and lyophilized to evaluate the specific weight after defined incubation time. Data were reported as mean values of measurements performed in triplicate.

### Mechanical Tests

Mechanical properties of scaffolds, before and after biomimetic treatment, were evaluated by compression tests, using a standard testing machine (Lloyd LR5K instrument, Fareham Hants, U.K.). The scaffolds (in triplicate) were soaked in dH_2_O until to obtain an equilibrium swelling, and then tested in a watery environment. The sample sizes were 5.8 ± 0.5 mm in height and 5.2 ± 0.2 mm in diameter. The deformation range and the speed (mm/min) were calculated, respectively, as 75 and 10% of swollen sample height. Young's modulus was calculated as the slope of the stress-strain curve.

### Morphological Investigation

Morphological analysis was carried out on scaffolds by using a Quanta FEG200 (FEI, the Netherland) Scanning Electron Microscope, in order to evaluate the porous structure and hydroxyapatite deposition. The measurements were performed on the surface and in cross-section after freeze-drying. For these investigations, the scaffolds were coated with gold (thickness of about 25–30 nm), by automatic sputter coater (EMSCOPE SC500, 20KV). X-ray energy dispersive spectroscopy (EDAX, Genesis 2000i) was also used to obtain a qualitative estimation of mineral deposits after biomimetic treatment.

### Attenuated Total Reflect Fourier Transform Infrared Spectroscopy (ATR FT-IR)

ATR FT-IR spectroscopy (Nicolet 5700) was implemented to identify the functional groups. Dried samples of the scaffolds before and after biomimetic treatment (5–7 mg) were scanned from 600 cm^−1^ to 2,000 cm^−1^ with a resolution of 2 cm^−1^ by using Thermo Fisher Nicolet IS10 (USA).

### *In vitro* Release Study

The *in vitro* peptide release profile from gelatin based scaffolds was studied by HPLC as reported in a previous study (Soriente et al., 2018). Briefly, bioactivated scaffolds were incubated in 200 μL sterile Tris-buffer solution (pH = 6.8) and kept in a shaking incubator (37°C, 40 rpm) at different time points. The supernatant was collected and same amount of fresh medium was added to each sample. 20 μL of supernatant was injected in a chromatograph equipped with an ultraviolet (UV) detector and a reversed phase column (Reprospher C18, 150 mm × 4.6 mm, DR. MAISCH, GmbH). The mobile phase systems consisted of 90% water and 10% acetonitrile. The flow rate was 1.0 mL/min and the wavelength was set at 220 nm. All experiments were triplicated for each sample.

### Biological Investigations

#### Cell Proliferation

The human mesenchymal stem cells (hMSCs) from adult bone marrow (purchased from Sigma-Aldrich, Italy) at 4th passage were cultured in α-Modified Eagle's medium (α -MEM) (Bio Whittaker, Belgium) containing 10% v/v FBS, 100 U/mL penicillin and 0.1 mg/mL streptomycin (HyClone, UK), in a humidified atmosphere at 37°C and 5% CO_2_.

The scaffolds before and after inorganic treatment and after organic functionalization were sterilized using 70% ethanol solution for 1 h and then in 1% antibiotic/antimycotic in PBS (2 h). hMSC (10,000 cells suspended in 20 μL of medium) were seeded in triplicate onto the scaffolds in a static condition. After 2 h of incubation to induce cell adhesion, scaffolds were moved in a new tissue culture plate and 1 mL of cell culture medium was added to each scaffold.

Scaffolds were treated with basal medium [α-MEM supplemented with 10 % Fetal Bovine Serum (FBS), antibiotic solution (streptomycin 100 μg/mL and penicillin 100 U/mL, Sigma Chem. Co.), and 2 mM L-glutamine] and maintained in culture for 21 days. The cell-culture medium was changed every 2–3 days.

Cell proliferation for scaffolds before and after inorganic treatment was analyzed by using Alamar Blue assay that quantifies cell metabolic activity. The cell–material constructs were removed from the culture plates at 1, 7, 14, and 21 days, washed with PBS (Sigma–Aldrich, Italy), and placed into a new plate. For each scaffold, DMEM medium without Phenol Red (HyClone, UK) containing 10% v/v Alamar Blue (AbD Serotec Ltd., Milano, Italy) was added. The samples were incubated for 4 h at 37°C and 5% CO_2_. 200 μL of solution were moved in a 96-well plate and the absorbance was measured using a spectrophotometer (VICTOR X3, PerkinElmer, Italy) at wavelengths of 540 and 600 nm. The cell viability is proportional to the magnitude of dye reduction and it is expressed as percentage of Alamar Blue reduction (Tabata and Ikada, 1998).

The cell proliferation for gelatin-based scaffolds bioactivated by BMP-2 peptide at different time (1, 3, and 7 days) was investigated by PicoGreen_dsDNA quantification kit (Invitrogen). This assay is based on the reaction of Picogreen dsDNA quantification reagent (100 μL) with cell lysates (100 μL) for 10 min in a 96-well plate. The fluorescence was assessed at a wavelength of 520 nm after excitation at 585 nm using a spectrophotometer. dsDNA was quantified according to a calibration curve of l-dsDNA standard in 10 mM Tris, 1 mM EDTA, pH 7.5, buffer. Each experiment was performed in triplicate.

#### Alkaline Phosphatase Assay

The alkaline phosphatase activity (ALP) of cells seeded onto scaffolds before and after inorganic treatment and organic functionalization was determined at different days of *in vitro* cell culture. To measure ALP levels, the cells were lysed in cell lysis buffer (BD Pharmingen™) and SensoLyte™ pNPP Alkaline Phosphatase Assay kit (AnaSpec, DBA, Milano, Italy) was used according to the manufacturer's instructions. The phosphatase activity was analyzed on 50 μL of cell lysates by measuring the activity of ALP enzyme which catalyses the cleavage of a phosphate group releasing p-nitrophenol from p-nitrophenyl phosphate in alkaline buffer solution after 1 h at 37°C. The absorbance was measured in a 96-well plate at 405 nm. The amount of ALP (nanograms) was calculated by a standard curve obtained using pNPP as substrate, and incubated at room temperature for 1 h. The DNA was quantified by employing PicoGreen_dsDNA quantification kit (Invitrogen) as described above.

The results were expressed as nanograms of ALP normalized to micrograms of DNA (ngALP/μgDNA).

### Statistical Analysis

Results were expressed as mean ± standard error of the mean (SEM) plotted on graph. Statistical analyses were performed by the GraphPad Prism software (version 7.0). Statistical significances between the time points were calculated using the two-way analysis of variance (ANOVA) test followed by Bonferroni's multi-comparison test.

## Results

### Swelling and Degradation Investigations of Neat Gelatin Scaffolds

**Figure 2:**
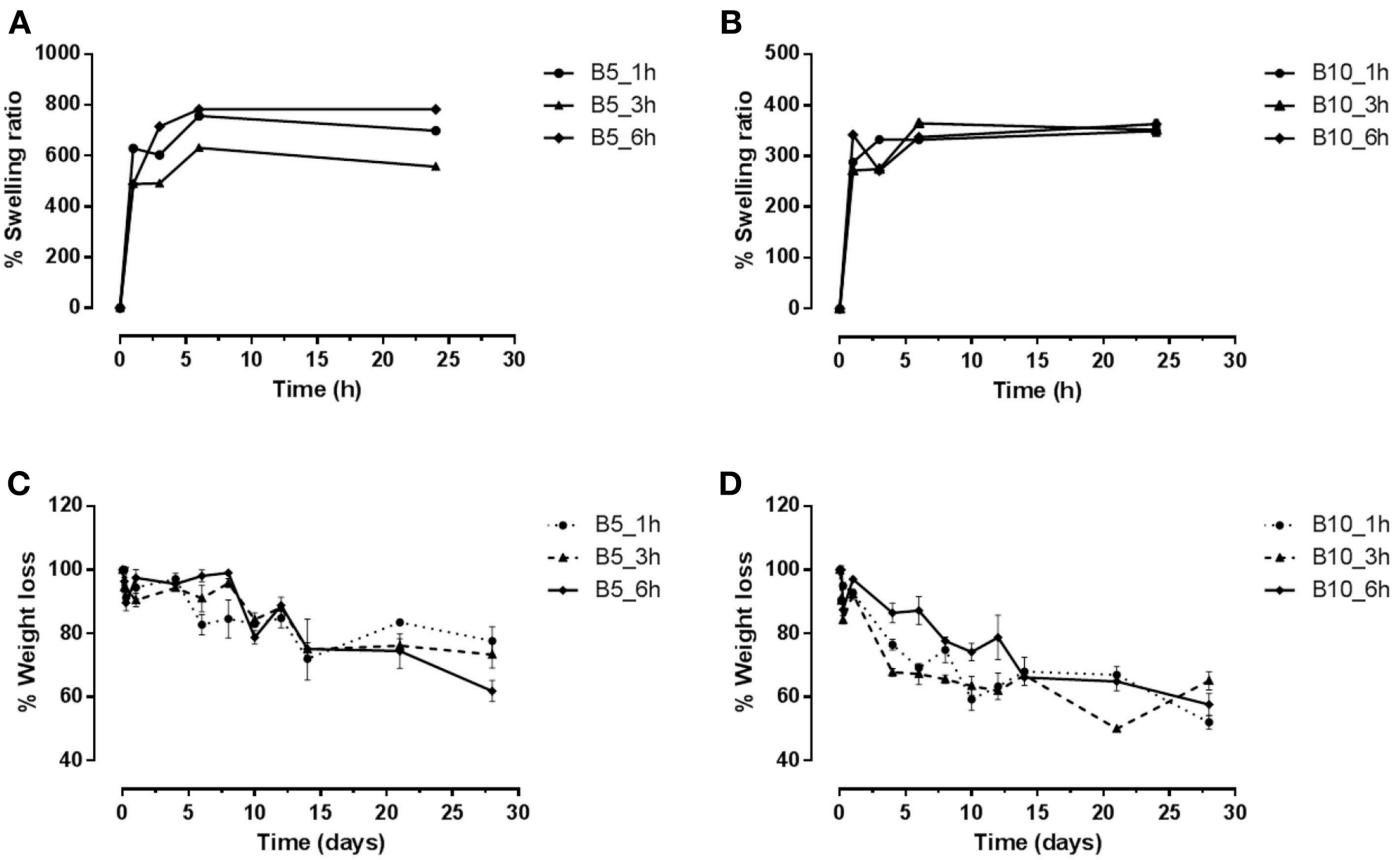
Diagrams of swelling and degradation of B5 **(A–C)** and B10 **(B–D)** scaffolds at different crosslinking times (1, 3, and 6 h).

**Figure 3:**
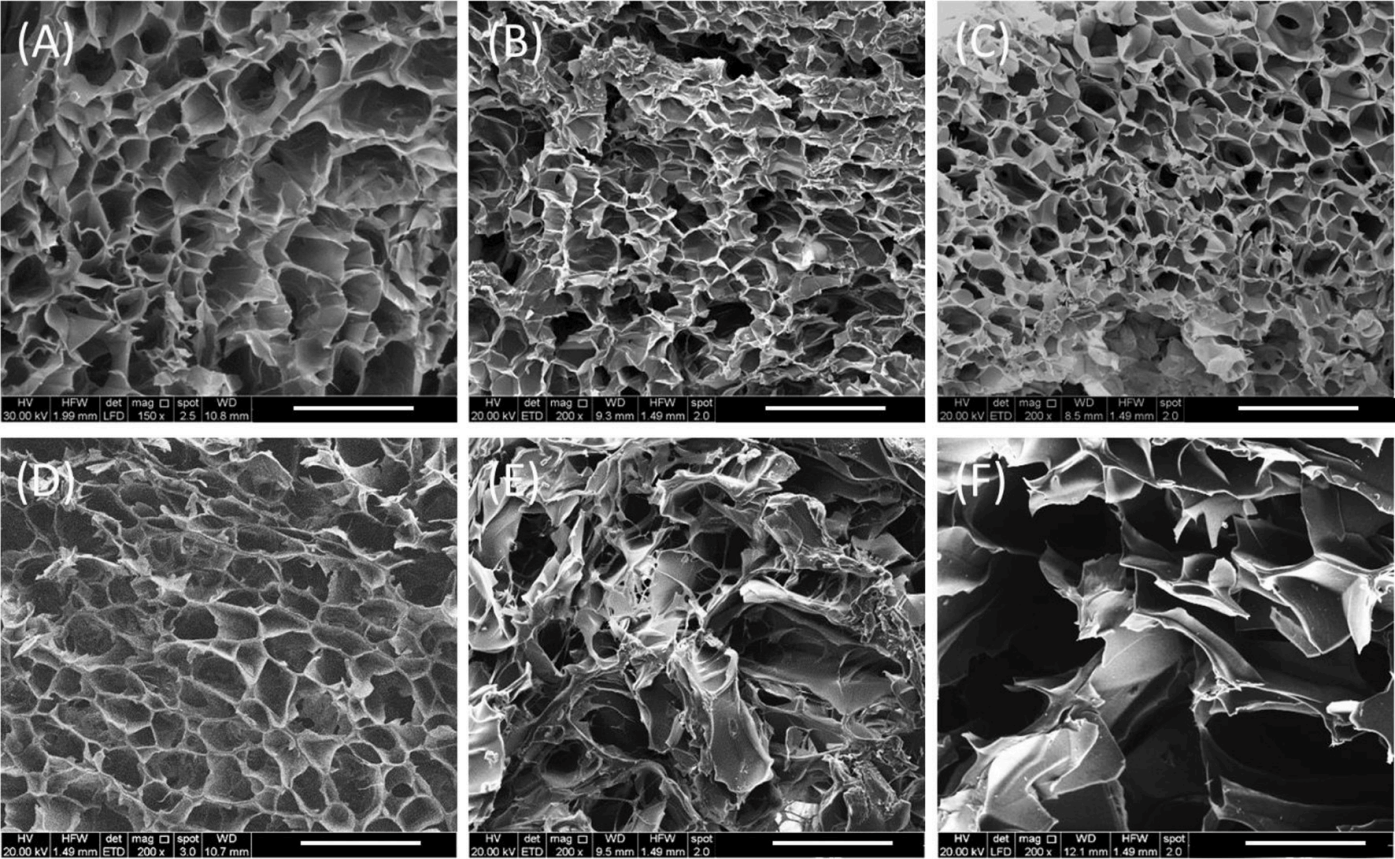
SEM images in cross-section of gelatin-based scaffolds crosslinked after 3 h. B5 scaffolds **(A–C)** and B10 scaffolds **(D–F)** at 0, 1, and 28 days of degradation, respectively. Scale Bars: 500 μm.

The swelling and degradation properties of type B gelatin at different concentrations (5–10 w/v%) and crosslinking time (1, 3, and 6 h) by gravimetric approach were evaluated. The swelling results (Figure 2) demonstrated that all samples reached the equilibrium after only 1–3 h. However, the swelling capability depends on gelatin concentration. Indeed, by increasing the gelatin amount, a reduction of swelling effect was observed. The B10 scaffolds showed, independently on the crosslinking parameters, a maximum of swelling ratio at about 350% (Figure 2B), while B5 scaffolds achieved a maximum at 600%, for B5_3h, and 700% for B5_1h and B5_6h (Figure 2A). This behavior may be mainly correlated to the different number of physical and chemical bonds in the structures. The hydration mechanism is based on a capillary phenomenon in which the water molecules penetrate the tiny interstices of triple-helical fibrils in the gelatin matrix. For this reason, the water penetration in samples with the lowest gelatin amount (B5) was easier than B10 samples where a higher density of physical crosslinking points was evident. Furthermore, the increased swelling capability of B5_6h may be probably ascribed to a EDC-induced degradation effect of gelatin at longer times (Cammarata et al., 2015). This behavior was also confirmed by the degradation study (Figures 2C,D). In particular, scaffolds with the lowest gelatin amount (B5) highlighted a slower degradation kinetic compared to B10 scaffolds. At day 28, B5 scaffolds (at 1 and 3 h of crosslinking time) showed a weight loss of about 24 wt%, while B5_6h showed a weight loss of 40 wt% (Figure 2C) (Cammarata et al., 2015). On the other hand, independently on crosslinking time, B10 scaffolds showed a weight loss of about 50 wt% at day 28 (Figure 2D). This different behavior among the two scaffolds groups (B5 and B10) may be ascribed to a good crosslinking efficacy obtained by a complete diffusion of EDC in each part of B5 scaffolds. This is also confirmed by the more homogeneous porosity as reported by the morphology study (Figure 3).

### Mechanical Test

Mechanical analysis has been performed on gelatin-based scaffolds (B5 and B10 at different crosslinking time) before and after biomimetic treatment. Results from unconfined compression test are summarized in Table 1. They highlighted the effect on the scaffold's mechanical behavior of: (i) polymer concentration, (ii) crosslinking time, and (iii) biomimetic treatment. With regard to the polymer concentration, B10 scaffolds showed significantly higher values of the Young's modulus if compared to B5 structures, before (*p* < 0.0001) and after (*p* < 0.001) biomimetic treatment. For the crosslinking time, by increasing the duration of the EDC treatment (from 1 to 6 h), mechanical performances did not change for B5 scaffolds, either before or after the inorganic functionalization, while B10 structures highlighted a different behavior. In particular, the Young's modulus slightly decreased by increasing the time, with or without the nucleation and crystallization of hydroxyapatite nanoparticles (*p* < 0.05). Different results among the scaffold groups before and after biomimetic treatment were detected. In particular, the hydroxyapatite nanoparticles seemed to negatively affect the mechanical features of B10 scaffolds, which showed decreased mechanical properties (*p* < 0.001). Meanwhile, treated B5 scaffolds did not show differences if compared to the untreated ones (Table 1).

**Table 1:** Results from mechanical analysis performed on gelatin scaffolds (B5, B10) at different crosslinking time (1, 3, and 6 h) before and after biomimetic treatment.

**Sample**	**No biomimetic treatment**	**Sample**	**Biomimetic treatment**
	**Young' s modulus (kPa)**		**Young' s modulus (kPa)**
B5_1h	195.2 ± 52.2	B5_1h/bio	139.0 ± 39.7
B5_3h	141.9 ± 7.2	B5_3h/bio	100.7 ± 18.5
B5_6h	123.8 ± 5.8	B5_6h/bio	88.5 ± 7.3
B10_1h	581.1 ± 148.1	B10_1h/bio	355.7 ± 99.7
B10_3h	512.8 ± 40.9	B10_3h/bio	285.8 ± 64.7
B10_6h	448.7 ± 43.6	B10_6h/bio	243.6 ± 23.8

*Data are expressed as main value ± standard deviation*.

### Morphological Analysis

#### Porosity and Structure

Morphological investigations highlighted that freeze-drying process allowed to obtain an interconnected porous network with a pore size of about 200 μm. That porosity should improve cell infiltration and growth, meanwhile the presence of smaller pores allows nutrients and body fluids transport. Furthermore, SEM images showed the change of porosity over the time (from 1 to 28 days) in wet conditions. In particular, in agreement with the results derived from the degradation tests, the morphological analyses highlighted that B5 scaffolds preserved their morphology from day 1 until day 28, while B10 scaffolds showed a different behavior (Figures 3A–C). The morphology changed in pore size and structure with an evident material loss as highlighted by the presence of great pores which appear at day 28 (Figures 3D–F).

#### Hydroxyapatite Nanoparticles Deposition by Biomimetic Treatment

**Scheme 1:**
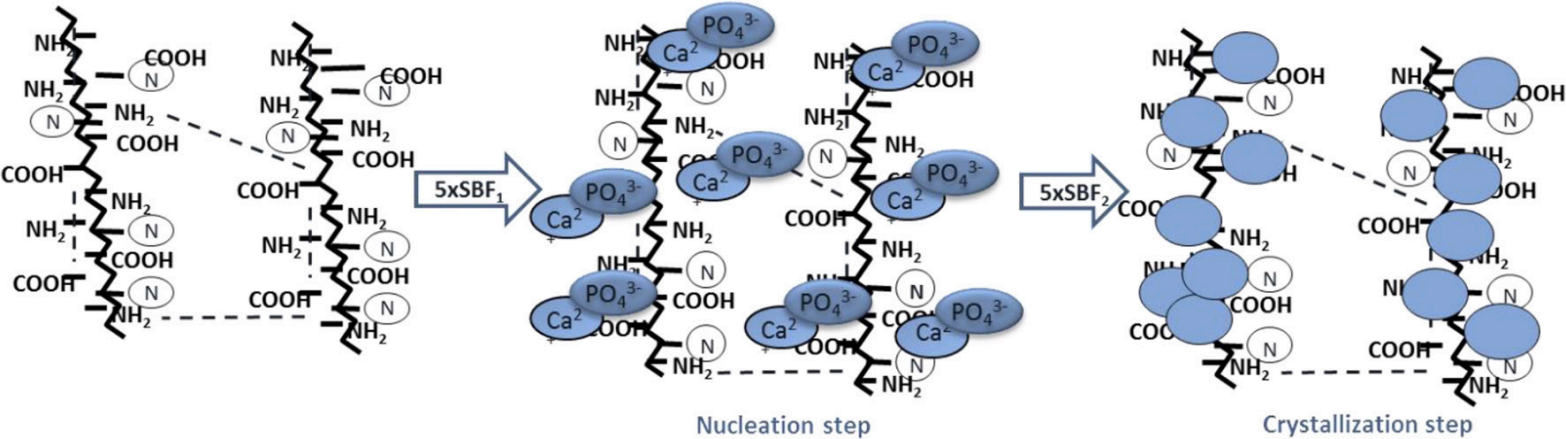
Biomimetic treatment by using supersaturated SBF solutions. Nucleation step obtained by incubation of samples in 5 × SBF1 for 3 days at 37°C pH = 6.5 followed by crystallization step by using a modified 5 × SBF2 for 4 days at 37°C pH = 6.0.

SEM and EDS analyses were performed on gelatin scaffolds biomimetically treated by SBF solutions for 7 days at 37°C. The treatment involves hydroxyapatite nucleation and crystallization steps as described in illustration scheme (Scheme 1). The nucleation step concerns the interaction between Ca^2+^ and ${\text{PO}}_{4}^{3-}$ ions with polymer chains followed by calcium phosphate nuclei formation. The second step involves the nuclei crystallization in hydroxyapatite nanoparticles (Scheme 1).

**Figure 4:**
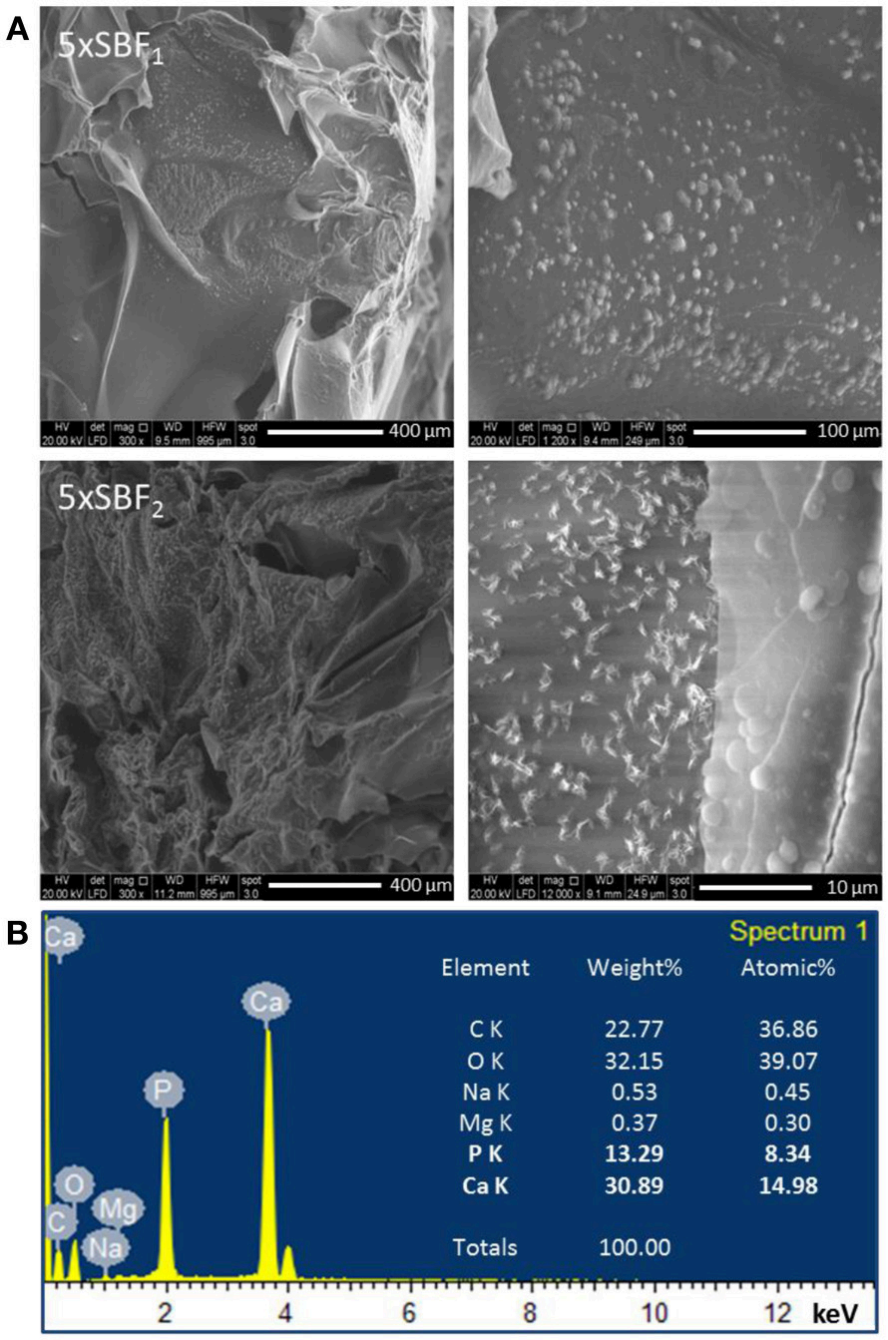
**(A)** SEM images of nuclei and hydroxyapatite deposits on B5_3h/bio scaffold after 7 days of SBF treatment. **(B)** EDS analysis of scaffold after 7 days of treatment time.

Moreover, SEM images (Figure 4A) of samples after 7 days of incubation time demonstrated the deposition of nanometric HAp on the internal pore walls, confirming that the high concentration of SBF solutions and a good porosity induced a fast and uniform deposition of hydroxyapatite in the scaffolds. The EDS analysis (Figure 4B) performed on the scaffold surface highlighted the presence of Ca and P ions with molar ratio (Ca/P = 1.79) approximately close to typical of hydroxyapatite (Ca/P = 1.67). The Ca/P ratio value is the result of a right balance of the Ca/P phases solubility which has been assured by controlling pH value between 6.5 and 6, respectively, during each treatment steps. Furthermore, some ions such as Na^+^ and Mg^2+^ which are common for biomimetic apatite were detected on the scaffold surface.

### Attenuated Total Reflect Fourier Transform Infrared Spectroscopy (ATR FT-IR)

**Figure 5:**
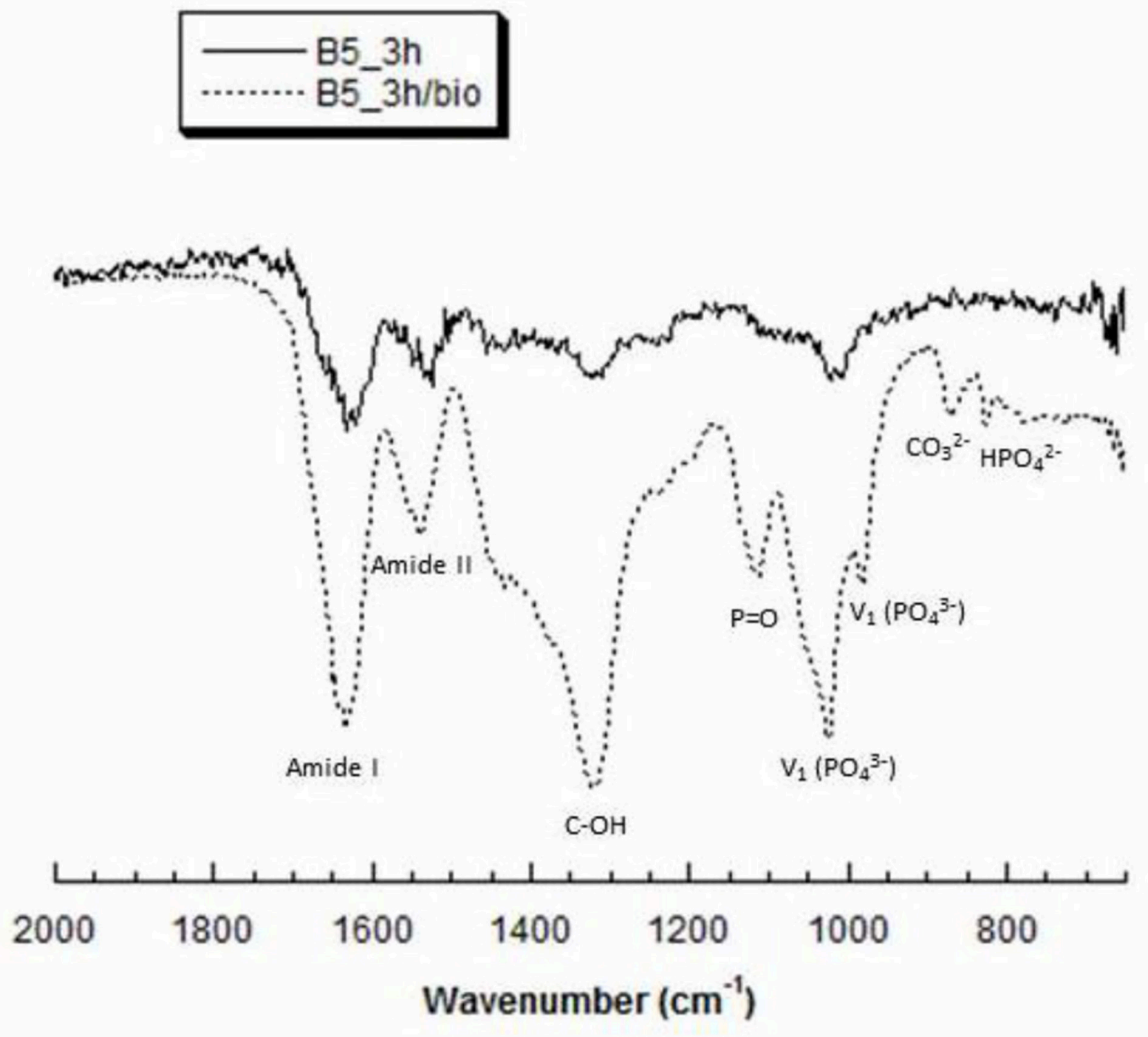
Representative ATR-FT IR spectra of B5 scaffolds, before (B5_3h) and after (B5_3h/bio) biomimetic treatment.

The ATR FT-IR spectrum (Figure 5) in the region of 1,750–800 cm^−1^ shows the main groups of hydroxyapatite and gelatin. In particular, the bands typical of gelatin B appear at 1,323 cm^−1^ which is attributed to vibration of proline side chains (Khan et al., 2012), 1,634 and ~1,540 cm^−1^ correspond to amide I and amide II modes which confirm the α-helical configuration of gelatin. Furthermore, phosphate bands appeared in the range 900–1,200 cm^−1^ with a typical peak of phosphate vibration at 1,025 cm^−1^. Meanwhile, the presence of ${\text{CO}}_{3}^{2-}$ and ${\text{HPO}}_{4}^{2-}$ bands demonstrated a non-stoichiometric HA.

### *In vitro* BMP-2 Peptide Release Study

**Figure 6:**
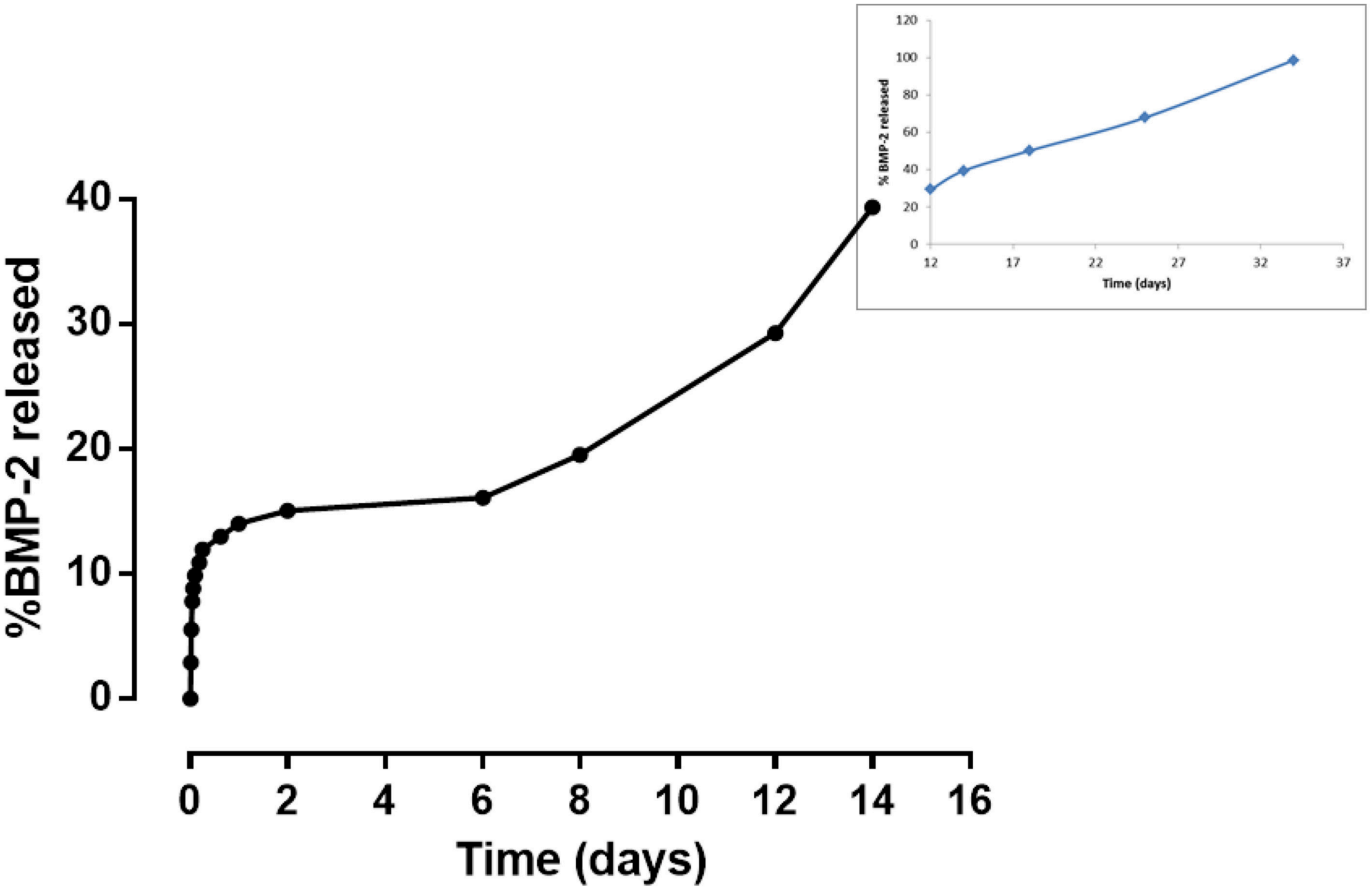
*In vitro* release study of BMP-2 peptide from gelatin-based scaffold at different time points.

The peptide synthesis by Fmoc solid phase route and by using microwave synthesizer allowed to obtain a peptide with high purity (>98%) with a molecular weight of 2041.3 Da as demonstrated by analytical HPLC and mass spectrometry, respectively (data not shown). The peptide was linked on gelatin based scaffolds by carbodiimide reaction mechanism (EDC:NHS), where the -COOH groups of gelatin and amide groups of BMP-peptide were involved. In this way, it was possible to obtain an *in vitro* peptide release for long time. Indeed, the results demonstrated a release of about 14% after 24 h, reaching a value of 40% after day 12 (Figure 6). However, the release is sustained up to 4 weeks where gelatin scaffold has released about 98% of peptide (Figure 6 in the frame).

### Biological Test

#### Effect of Biomimetic Treatment on Cellular Behavior: Proliferation and ALP Expression

**Figure 7:**
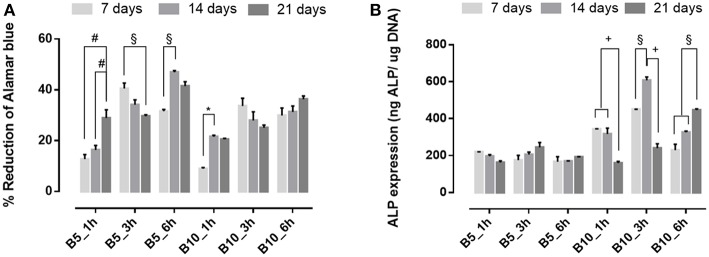
**(A)** Alamar blue results and **(B)** Alkaline phosphatase expression obtained from hMSC seeded on scaffolds before SBF treatment at 7, 14, and 21 days of culture time. ^*^p ≤ 0.05, §*p* ≤ 0.01, +*p* ≤ 0.001, and #*p* ≤ 0.0001, for each group at 7, 14, and 21 days.

**Figure 8:**
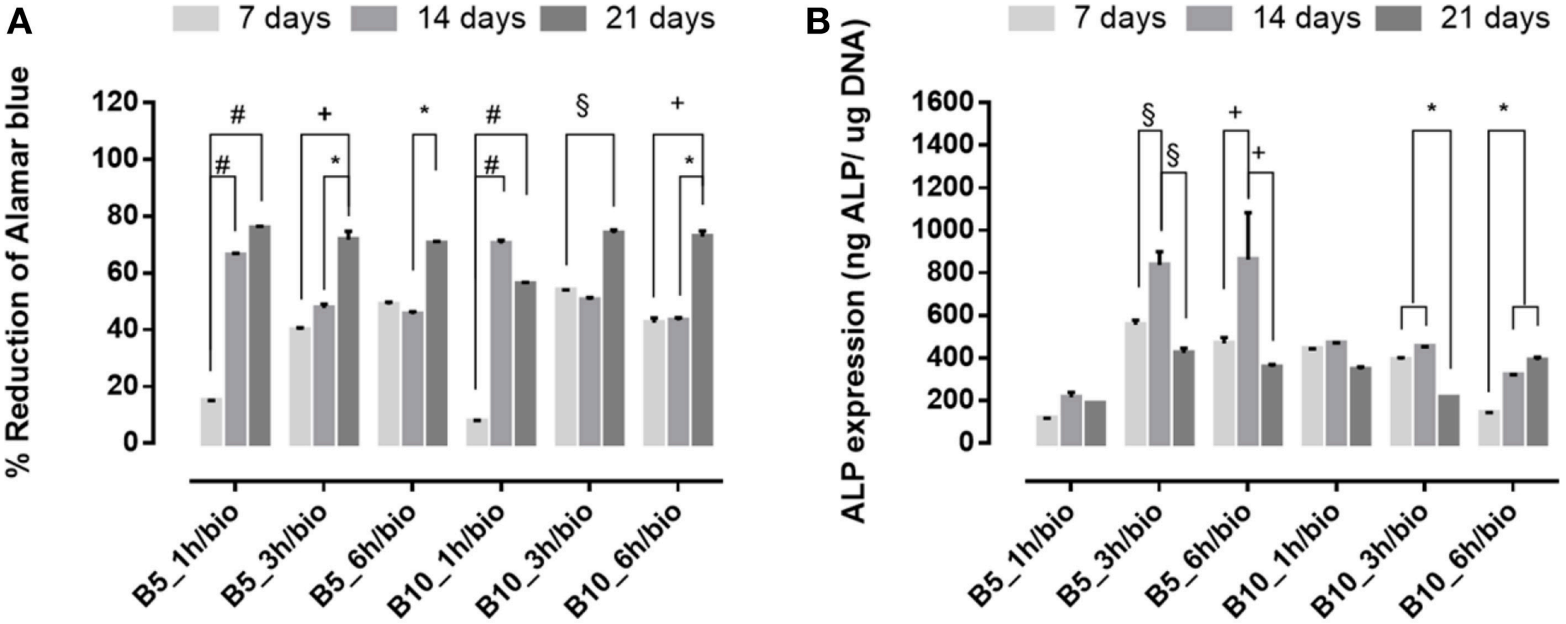
**(A)** Alamar blue results and **(B)** Alkaline phosphatase expression obtained from hMSC seeded on scaffolds after SBF treatment at 7, 14, and 21 days of culture time. ^*^p ≤ 0.05, §*p* ≤ 0.01, +*p* ≤ 0.001, and #*p* ≤ 0.0001, for each group at 7, 14, and 21 days.

To evaluate the effect of biomimetic treatment on proliferation and osteogenic differentiation of hMSC, cells were seeded on gelatin scaffolds before and after biomimetic treatment. The biological results showed a different cellular behavior in terms of cell proliferation between material groups (B5 and B10) before (Figure 7A) and after SBF treatment (Figure 8A). In detail, before biomimetic treatment the cells seeded on B5 scaffolds showed higher cell proliferation values than B10 group (Figure 7A). However, B10 scaffolds before SBF treatment showed higher ALP expression than B5 (Figure 7B). This different behavior between B5 and B10 material groups may be ascribed to more compact structure in B10 which improved the structural properties. It's well-demonstrated the effect of matrix stiffness on the phenotype and differentiation pathway of MSCs (Pek et al., 2010).

An opposite behavior was observed after biomimetic treatment (Figure 8). Indeed, cell proliferation increased over culture time for both B5 and B10 samples (Figure 8A), while higher ALP levels were achieved only for B5 scaffolds (Figure 8B). The best behavior was observed for B5 scaffolds after 3 and 6 h of crosslinking time. The ALP peak was observed in a basal condition without osteogenic factors (i.e., ascorbic acid, B-dexamethasone, B-glycerophosphate) at day 14, thus confirming that the presence of solid bioactive signals on surface is important for the expression of early marker of osteogenic differentiation as ALP.

#### Effect of BMP-2 Peptide on Cellular Behavior

**Figure 9:**
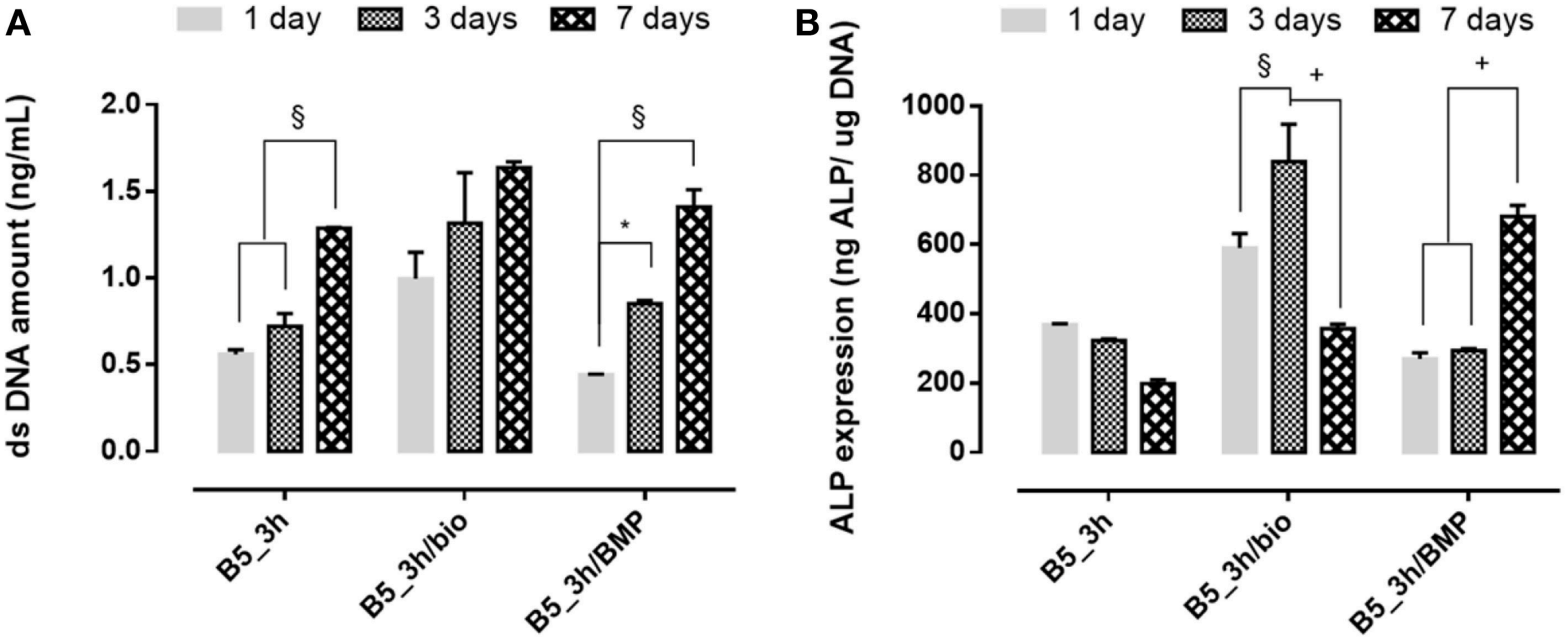
**(A)** dsDNA amount results and **(B)** ALP expression of functionalized scaffolds at 1, 3, and 7 days of *in vitro* cell culture. ^*^p ≤ 0.05, §*p* ≤ 0.01, and +*p* ≤ 0.001, for each group at 7, 14, and 21 days.

The effect of covalent immobilization of BMP-2 peptide on B5 scaffolds in terms of cell proliferation and early osteogenic differentiation of hMSC was investigated (Figure 9). The results demonstrated that cells better adhered and proliferate (*p* ≤ 0.01) on scaffolds after biomimetic treatment (B5_3h/bio) and no significative effect of functionalization on cell proliferation in terms of DNA amount at different time points (1, 3, and 7 days) between neat B5 scaffolds (B5_3h) and BMP-2 functionalized ones (B5_3h/BMP) was observed (Figure 9A). However, different behavior for ALP expression was evaluated. The highest ALP value was detected for B5_3h/bio at day 3, meanwhile the covalent immobilization of BMP-2 peptide allowed to obtain the highest ALP level at longer time (Figure 9B).

## Discussion

Tissue regeneration process needs of an appropriate 3D structure with specific performances able to promote cell adhesion and proliferation, leading to new tissue formation. Several methods have been proposed to improve the cell recruitment and growth (Tanahashi et al., 1992; Russo et al., 2015; Ronca et al., 2017; D'Amora et al., 2018a). Here, it was investigated the possibility to functionalize, at nanoscale level, gelatin-based scaffolds by two different approaches to control the biological response. The functionalization by biomimetic method and analog of BMP-2 peptide was performed on crosslinked scaffolds by carbodiimide route [i.e., 1-Ethyl-3-(3-dimethylaminopropyl) carbodiimide, EDC]. The chemical crosslinking was performed at different times (1, 3, and 6 h) to evaluate its effect on physico-chemical and mechanical properties. In particular, EDC solution was used to bind amino acidic functional groups in order to have an intramolecular and short-range intermolecular bonds (amine bond), with some pendant side branches (N-acyl urea groups). Crosslinking treatment creates new stable chemical bonds in the hydrogel structure, limiting the chains mobility. The concept of chains mobility, for a crosslinked and not crosslinked hydrogel, is related to its swelling/deswelling capability; it depends on physical (entanglements) and chemical (hydrogen and/or amine) bonds. Indeed, B10 scaffolds showed lower chain mobility than B5 leading to lower swelling and faster degradation behavior. Furthermore, results demonstrated that for B10 scaffolds the effect of crosslinking time was not observed, while for B5 scaffolds, an increasing of swelling and degradation behavior at the highest crosslinking time (B5_6h) were detected. The fast degradation for B5_6h was due to the detrimental effect of crosslinker at longer time on the polymer structure (Cammarata et al., 2015). The effect of polymer composition (B5 and B10) and crosslinking time influences the mechanical properties. The increase in gelatin amount improves scaffold stability in terms of Young's modulus due to higher polymer density and lower water amount. The same trend was also assessed between B5 and B10 scaffold groups after biomimetic treatment. Furthermore, after biomimetic treatment, only B10 scaffolds showed decreased mechanical properties probably due to combined effects of EDC-induced degradation and poor crosslinking efficiency of more concentrated gelatin (Cammarata et al., 2015). Meanwhile, treated B5 scaffolds did not show differences if compared to the untreated ones. Physico-chemical characterization (i.e., ATR FT-IR) demonstrate the presence of hydroxyapatite and dicalcium phosphates on the scaffold materials as also evident by SEM images. Hydroxyapatite nanoparticles were well-distributed on surface and inside of scaffolds acting as bioactive cue for cellular behavior. Indeed, an improvement of cell proliferation over culture time was observed for all biomimetic samples. Furthermore, an higher early ALP expression was also evaluated for treated B5 scaffolds than B10. This behavior may be correlated to the swelling properties. B5 scaffolds showed higher swelling performances than B10 and this behavior allowed higher adsorption of ions in SBF solution. Based on physico-chemical, morphological and mechanical properties, the best behavior was observed for B5 after 3 h of crosslinking time (B5_3h). The second approach to improve the bioactivity of scaffold was the organic decoration by BMP-2 peptide covalently linked to the B5 scaffold material (B5_3h). The kinetic release study demonstrated that covalent linking between -COOH groups of gelatin and amide groups of BMP-peptide allows to obtain an *in vitro* peptide release up to 4 weeks. In particular, after 12 days the peptide release achieved 40 % followed by a slowly release reaching 98% after 4 weeks. This results had effect on biological properties concerning the influence of peptide bioactivation on cellular behavior. In particular, in terms of cell proliferation measured by the dsDNA amount, it was observed that the biomimetic treatment (B5_3h/bio) allowed the improvement of cell-material interaction if compared to neat (B5_3h) and organic functionalized scaffold (B5_3h/BMP). This behavior may be explained by an increase in surface roughness which improved the attachment and proliferation over culture time. Furthermore, the inorganic signal induced an early ALP expression at short time. On the contrary, B5_3h/BMP showed higher ALP value at day 7 due to the availability of BMP-2 peptide that achieved the optimal concentration in cell culture medium at day 7 so triggering the cell differentiation mechanism (Soriente et al., 2018). However, the present work suggested that both bioactivated scaffolds, with inorganic and organic signals, were able to guide osteoinductive processes through the expression of early markers of osteogenesis at different time.

## Conclusions

This study reported the development and bio-functionalization of gelatin-based scaffolds by using two different approaches: inorganic and organic bioactive signals decoration. The scaffold composition and crosslinking time influenced the scaffold performances in terms of physico-chemical, morphological and mechanical behavior. Furthermore, both bioactive signals were able to improve *in vitro* biological activities at different time. In particular, biomimetic approach improved cell attachment and early osteogenic differentiation at short time, meanwhile BMP-2 peptide decoration operated *in vitro* as bioactive signal at long time. Indeed, covalent linking of peptide on gelatin scaffold by carbodiimide route allowed to obtain *in vitro* release at long time, so influencing the cellular behavior in terms of early osteogenic differentiation.

However, these two approaches allowed to study the possibility to functionalize at nanoscale level polymeric scaffolds by tuning the biological response at short and long time of human undifferentiated mesenchymal cells.

## Author Contributions

MR and UD: conceptualization. MR, AR, UD, and CD: methodology. AR, UD, MR: validation. AR, UD, MR, and CD: investigation. AR, UD, and MR: writing and original draft preparation. LA: supervision and funding acquisition.

### Conflict of Interest Statement

The authors declare that the research was conducted in the absence of any commercial or financial relationships that could be construed as a potential conflict of interest.
